# Changes in emergency department visits and mortality during the COVID-19 pandemic: a retrospective analysis of 956 hospitals

**DOI:** 10.1186/s13690-023-01234-9

**Published:** 2024-01-12

**Authors:** Mahya Razimoghadam, Mehdi Yaseri, Mohammad Effatpanah, Rajabali Daroudi

**Affiliations:** 1 Department of Health Management, policy and Economics, School of Public Health, Tehran University of Medical Sciences, Tehran, Iran; 2 Department of Epidemiology and Biostatistics, School of Public Health, Tehran University of Medical Sciences, Tehran, Iran; 3 Pediatric department, School of Medicine, Imam Khomeini hospital, Tehran University of Medical Sciences, Tehran, Iran; 4 National Center for Health Insurance Research, Tehran, Iran

**Keywords:** COVID-19, Hospital emergency service, Substance-related disorders, Mental disorders, Cardiovascular diseases, Self-injurious behavior

## Abstract

**Background:**

During the COVID-19 pandemic, many non-COVID-19 emergency department (ED) visits were indirectly affected. ED visits and mortality were assessed during different pandemic time periods compared with pre-pandemic.

**Methods:**

The study used data from 41 million Iran Health Insurance Organization members. The outcomes were non-COVID-19 ED visits and associated mortality in 956 hospitals. An analysis of ED visits was conducted both for all-cause and cause-specific conditions: cardiovascular diseases (CVD), mental and substance use disorders, unintentional injuries, and self-harm. In addition, total in-hospital ED mortality was analyzed. A negative binomial regression and a Poisson regression with a log link were used to estimate the incidence rate ratio (IRR) of visits and mortality relative risk (RR).

**Results:**

1,789,831 ED visits and 12,377 deaths were reported during the study. Pre-pandemic (Sep 2019 to Feb 2020), there were 2,767 non-COVID-19 visits rate per million person-month, which decreased to 1,884 during the first COVID-19 wave with a national lockdown from Feb 20 to Apr 19, 2020 (IRR 0.68, [0.56–0.84]). The non-COVID-19 ED mortality risk was 8.17 per 1,000 visit-month during the pre-pandemic period, rising to 12.80 during the first wave of COVID-19 (RR 1.57, [1.49–165]). Non-COVID-19 ED visit rates decreased during the first pandemic year from Sep 2020 to Feb 2021 (IRR 0.73, [0.63–0.86]), but increased after COVID-19 vaccination two years later from Sep 2021 to Feb 2022 (IRR 1.11, [0.96–0.17]). The total ED mortality risk for non-COVID-19 was significantly higher after the COVID-19 outbreak in the first (RR 1.66, [1.59–1.72]) and second years (RR 1.27, [1.22–1.32]) of the pandemic. The visit incidence rate for mental health and substance use disorders declined from 8.18 per million person-month to 4.57 (IRR 0.53, [0.32 to 0.90]) in the first wave. In the second year, unintentional injury visits increased significantly compared with pre-pandemic (IRR 1.63, [1.30–2.03]). As compared to before the pandemic, there was no significant change in CVD and self-harm visit rates during the pandemic. Cardiac arrest was the leading cause of death in Iran hospitals’ EDs.

**Conclusion:**

In the first year of the COVID-19 pandemic, non-COVID-19 hospital ED visits declined and mortality risk increased. Despite two years since the COVID-19 outbreak, non-COVID-19 ED mortality risk remains high.

**Table:** 

**Text box 1. Contributions to the literature**
• COVID-19 indirect effects on non-COVID-19 hospital ED visits and associated mortality investigated in Iran.
• Due to improved Iranian electronic health record databases, this study uses large data sets for 41 million Iranians across 956 hospitals.
• An analysis was conducted both for all-cause and cause-specific conditions: cardiovascular diseases, mental and substance use disorders, unintentional injuries, and self-harm.
• In the first wave of COVID-19, the total non-COVID-19 visit rate decreased. Although this decrease persisted in the first year of the pandemic, visit rates increased in the second year after COVID-19 vaccination.
• During all the periods after the COVID-19 outbreak, the total non-COVID-19 mortality risk was significantly higher than pre-pandemic.

## Background

Coronavirus disease 2019 (COVID-19) severely impacts global health systems. The World Health Organization (WHO) declared COVID-19 a pandemic on March 11, 2020 [1]. During the COVID-19 pandemic, health systems were challenged to provide timely and adequate healthcare. At the beginning of the pandemic, many countries announced difficulties in providing non-COVID-19 services [2–9]. Health services disruptions can be caused by supply interruptions or demand decreases. In terms of service demand, fear of contracting the SARS-CoV-2 virus in healthcare facilities, especially hospitals, long waiting times, the misconception that centers were closed, as well as the economic hardships that plagued people during the pandemic were among the most significant factors [10, 11]. A number of disruptions in the supply of health care services have been attributed to the non-provision of elective services, overworked medical personnel, inadequate beds and medical equipment, the closure of diagnostic and outpatient clinics, as well as changes in treatment policies [12, 13]. In most countries, service supply side factors have had a greater impact than service demand side factors, according to WHO reports [12–14].

The changes that have occurred as a result of the COVID-19 pandemic in the provision and consumption of health services by non-COVID-19 patients are referred to as the indirect or collateral effects of the pandemic [15]. The extent of these indirect effects varies depending on differences among patients, variations in healthcare services, and the approaches taken to manage the COVID-19 pandemic in different countries. These indirect effects, such as changes in lifestyles, unavailability of services, or postponement of services, will have consequences for people’s health [15–18]. These consequences can include not only affecting mortality rates but also resulting in future morbidity. In low- and middle-income countries, the indirect effects of the COVID-19 pandemic on non-COVID-19 health conditions tended to be worse [15, 19, 20]. It is still important to investigate COVID-19 indirect effects even after more than three years since the virus outbreak. Over time, lockdowns, healthcare disruptions and lifestyle changes have revealed their long-term indirect effects [14, 17, 19]. Many researchers have examined the indirect effects of the COVID-19 pandemic on hospitalization, and health outcomes among people with non-COVID-19 diseases [21–27]. Hospital Emergency Departments (ED) were investigated as the front line of care. Many studies reported that ED visits decreased significantly, particularly avoidable visits [15, 18, 28–38]. In particular, ED visits due to cardiovascular, mental disorders, and self-harm changed during the COVID-19 pandemic [29, 39–48]. The COVID-19 pandemic has contributed to an increased risk of cardiovascular disease, fueled by reduced physical activity, sedentary lifestyles, heightened stress levels, and the potential impact of SARS-CoV-2 infection on individuals with pre-existing heart conditions [17, 19, 49]. Conversely, the impact of quarantine measures, social distance protocols, unemployment rates, and economic pressures has resulted in a surge in mental health disorders [19, 50–59]. These circumstances have contributed to the spread of substance abuse, alcohol-related health issues, and self-harm tendencies among individuals [36, 42, 44, 47, 60]. Numerous studies indicate that the pattern of injuries, including unintentional injuries, has significantly changed during the COVID-19 pandemic, primarily due to changes in work conditions and restrictions on travel [43, 61–63].

Iran was among the first countries worldwide to be affected by COVID-19 virus outbreaks [64, 65]. Iranian officials confirmed the first positive SARS-CoV-2 infection on February 19, 2020. As of November 2023, over seven million people have been infected with COVID-19 in Iran, and over 146,000 people have died as a result [66]. Iran faced some challenges from the beginning of the COVID-19 pandemic [67, 68]. Several factors contributed to this, including a lack of Personal Protective Equipment (PPE), ICU beds, and trained medical staff [69, 70]. According to estimates, between 5% and 10% of nurses in various hospital sections are assigned to dealing with COVID-19 cases [67]. Consequently, these challenges underscore the urgent requirement to prioritize non-COVID-19 health conditions assessments within Iranian hospitals. In addition, the emergency department plays an important role in providing affordable healthcare 24 hours a day, seven days a week in Iran. Moreover, the primary healthcare system in Iran faces several challenges, including a lack of flexibility, effectiveness, readiness, and fair distribution of resources [71–73]. These challenges have a significant impact on its overall performance. Underperforming primary health care systems have a direct impact on hospital emergency department (ED) admissions, especially during crises [74–77]. Therefore, it is important to examine the effects of the COVID-19 pandemic on ED admission characteristics in Iran.

To date, no studies have been conducted regarding the changes in non-COVID-19 ED visits during the pandemic to show whether Iran experienced the indirect or collateral effects of the pandemic. For this study, we examined the changes in all-cause ED visits and related mortality from before the pandemic to different periods afterwards. To conduct a thorough investigation, a cause-specific analysis was performed on the rate of admissions in the ED, focusing on selected acute health conditions considered most impacted during the COVID-19 pandemic. The analysis spans pre-pandemic to two years after the pandemic to shed light on pandemic circumstances, including healthcare access delay, on non-COVID-19 outcomes.

## Method

### Data source

This study is retrospective and cross-sectional. The claim data required were received from the Statistics and Information Technology Center of the Iran Health Insurance Organization (IHIO). This center collects hospital claim information from 41 million IHIO members admitted to 956 hospitals. Data was collected from hospital ED registrations and discharge statuses. The claims data examined in this study only included primary diagnosis codes and didn’t include patient triage information.

### Study period

This study was conducted using seventeen months of extracted data, spanning four different time periods. The first period was before the COVID-19 pandemic in Iran, from September 23, 2019, to February 19, 2020. The second period was related to the first COVID-19 wave in Iran, which was accompanied by a nationwide lockdowns from February 20 to April 19, 2020. During this period, national lockdowns and full restrictions were implemented in the country to prevent COVID-19 spread. The third and fourth time periods were chosen one and two years after the pandemic and in the same months as the pre-pandemic period. The third period was post-pandemic and ran from September 22, 2020 to February 18, 2021. The fourth period was from September 23, 2021, to February 19, 2022, after nationwide vaccine availability. During that period, 40% of Iran’s population received at least one dose of the COVID-19 vaccine, called the post-vaccination period.

### Health outcomes

The primary outcome of this study is a hospital ED visit. Separate analyses are presented for each age, gender, and selected health condition. All-cause and cause-specific admission rates are estimated. The secondary outcome of the study is all-cause mortality, which is defined as “death” in the ED discharge status. Mortality is not tracked after discharge or in other hospital sections. Birth determination classifies gender as male and female. Age groups include 17 and under, 18–44, 45–64, and over 64. These categories are based on the age classification of pediatrics, young adults, middle aged and older adults [78, 79]. A three-digit International Classification of Diseases, tenth revision, was used to diagnose health conditions. There was no information about the details of patient triage information in the ED in the claims data examined in this study. Based on the literature, the conditions were selected depending on national importance and those that might be most affected by the COVID-19 pandemic in EDs. Selected health conditions include cardiovascular diseases, mental and substance use disorders, unintentional injuries, and self-harm. The U07 codes associated with registered COVID-19 patients were excluded from the study and are only shown schematically in the ranking of frequent diagnoses.

### Statistical analysis

The ED visit incidence rate was calculated by dividing the number of ED visits by the number of IHIO members during that time period and was reported per million person-month. The mortality risk was measured as the number of cases discharged by “death” status minus the number of ED visits during that time period. Mortality risk is reported per 1,000 visit-month.

Comparing pandemic periods to pre-pandemic, a Poisson regression or negative binomial regression was applied based on the presence of overdispersion [80]. Negative binomial regression was used to estimate the Incidence Rate Ratio (IRR) to compare ED visit incidence rate per million person-month in aggregated data. We considered the pre-pandemic period as the baseline, and ED visit incidence rates in the first wave, post-pandemic and post-vaccination period compared to pre-pandemic. Total IHIO members were used as exposure variables to estimate overall unadjusted IRRs. However, to estimate IRRs by age and gender, IHIO members were entered as exposure variables within their respective age and gender groups. To compare ED mortality risks of first wave, post-pandemic and post-vaccination with pre-pandemic baseline, a Poisson regression with a log link was used. Based on binary mortality data, Poisson regression estimates the Relative Risk (RR). No offset was added to the regression analysis. Statistical analyses were executed with Stata Statistical Software Release 17 College Station, TX: StataCorp LLC. IRRs and RRs are reported with 95% confidence intervals. A significance level of 0.05 was used for statistical analysis.

## Results

### All-cause ED visits and mortality

A total of 1,789,831 non-COVID-19ED visits were recorded during the seventeen months of the study. During the pre-pandemic period, 114,355 monthly ED visits were registered, which reached 78,055 monthly visits during the first wave of COVID-19. The monthly number of visits during the post-pandemic period was 84,384, and during the post-vaccination period 128,005.

A total of 12,377 deaths associated with non-COVID-19 diseases was recorded in the ED during the study period. Before the pandemic, there were 934 deaths in the ED per month. Monthly deaths were 999 during the first wave and 1,142 during the post-pandemic period. After vaccination, deaths reached 1,329 per month. As compared to other age groups, elderly people above 64 have the highest number of ED deaths. Table 1 presents details about ED visits and related mortality.

**Table 1:** Emergency department visits and mortality descriptions during the COVID-19 pandemic

Time periods	Pre-pandemic (Sep2019-Feb 2020)		1st wave (Feb2020-Apr 2020)		Post-pandemic (Sep2020-Feb 2021)		Post-vaccination (Sep2021-Feb 2022)	
Duration (months)	5		2		5		5	
		Percent (%)		Percent (%)		Percent (%)		Percent (%)
**ED visit (number)**	571,773	100.0	156,110	100.0	421,922	100.0	640,026	100.0
Age groups								
< 18	92,754	16.2	20,695	13.3	53,871	12.8	83,511	13.0
18–44	189,597	33.2	56,685	36.3	141,162	33.5	220,323	34.4
45–64	146,624	25.6	41,556	26.6	118,853	28.2	184,564	28.8
< 64	142,798	25.0	37,174	23.8	108,036	25.6	151,628	23.7
Gender								
Female	281,309	49.2	73,748	47.2	203,573	48.2	314,087	49.1
Male	290,464	50.8	82,362	52.8	218,349	51.8	325,939	50.9
Selected health conditions								
Cardiovascular diseases	15,786	2.8	4,460	2.9	14,156	3.4	17,663	2.8
Mental and substance use disorders	1,493	0.3	328	0.2	992	0.2	1,955	0.3
Unintentional Injuries	1,832	0.3	834	0.5	1,980	0.5	2,994	0.5
Self-Harm	209	0.0	102	0.1	286	0.1	345	0.1
**ED Mortality (number)**	4,671	100.0	1,998	100.0	5,708	100.0	6,646	100.0
Age groups								
< 18	229	4.9	94	4.7	222	3.9	305	4.6
18–44	401	8.6	177	8.9	546	9.6	693	10.4
45–64	1,125	24.1	520	26.0	1,509	26.4	1,881	28.3
> 64	2,916	62.4	1,207	60.4	3,431	60.1	3,767	56.7
Gender								
Female	1,929	41.3	824	41.2	2,252	39.5	2,615	39.3
Male	2,742	58.7	1,174	58.8	3,456	60.5	4,031	60.7

The incidence rate of non-COVID-19 ED visits before the pandemic was 2,767 per million person-month, which decreased to 1,884 during the first wave (IRR 0.68, [0.56–0.84]), and the greatest decrease was among those under eighteen years of age (IRR 0.56, [0.40–0.79]). The incidence rate of ED visits after the pandemic was 2,031 per million person-month, a decrease from the pre-pandemic rate (IRR 0.73, [0.63–0.86]). During the post-vaccination period, the visit incidence rate was 3,081 per million person-month (IRR 1.11, [0.96–0.17]). Table 2 provides more information about the ED visit incidence rate and IRR for each time period.

**Table 2:** Non-COVID-19 incidence rate of emergency department visits (per million person-month) and Incidence Rate Ratio (IRR) of COVID-19 pandemic compared to the pre-pandemic baseline

Time periods	Pre-pandemic (Sep 2019-Feb 2020)	First Wave (Feb 2020-Apr 2020)					Post-pandemic (Sep 2020-Feb 2021)					Post-vaccination (Sep 2021-Feb 2022)				
	Incidence Rate (Reference)	Incidence Rate	IRR	Confidence Interval		p-value	Incidence Rate	IRR	Confidence Interval		p-value	Incidence Rate	IRR	Confidence Interval		p-value
Total	2766.86	1884.44	0.68	0.56	0.84	0.00	2030.98	0.73	0.63	0.86	0.00	3080.85	1.11	0.96	1.30	0.17
< 18	1650.59	925.28	0.56	0.40	0.79	0.00	969.65	0.59	0.46	0.76	0.00	1503.16	0.91	0.72	1.15	0.44
18–44	1975.47	1480.05	0.75	0.62	0.91	0.00	1478.95	0.75	0.66	0.84	0.00	2308.32	1.17	1.05	1.30	0.01
45–64	3763.01	2631.29	0.70	0.56	0.88	0.00	2952.14	0.78	0.68	0.90	0.00	4584.31	1.22	1.07	1.39	0.00
< 64	9203.31	5850.50	0.64	0.51	0.79	0.00	6556.12	0.71	0.64	0.80	0.00	9201.48	1.00	0.90	1.11	1.00
Gender																
Female	2804.95	1833.49	0.65	0.47	0.92	0.01	2016.70	0.72	0.55	0.93	0.01	3111.51	1.11	0.86	1.43	0.43
Male	2730.94	1932.56	0.71	0.56	0.89	0.00	2044.48	0.75	0.64	0.88	0.00	3051.88	1.12	0.95	1.32	0.18
Health Conditions																
Cardiovascular diseases	167.59	117.52	0.70	0.35	1.41	0.32	140.19	0.89	0.53	1.50	0.67	168.17	1.11	0.67	1.84	0.68
Mental and substance use disorders	8.18	4.57	0.53	0.32	0.90	0.02	4.75	0.64	0.43	0.97	0.04	9.41	1.27	0.86	1.88	0.23
Unintentional Injuries	10.19	11.49	1.14	0.82	1.57	0.44	10.47	1.08	0.84	1.38	0.57	16.65	1.63	1.30	2.03	0.00
Self-Harm	0.92	1.11	1.16	0.55	2.46	0.70	1.28	1.24	0.74	2.08	0.41	1.44	1.59	0.94	2.70	0.09

**Fig. 1:**
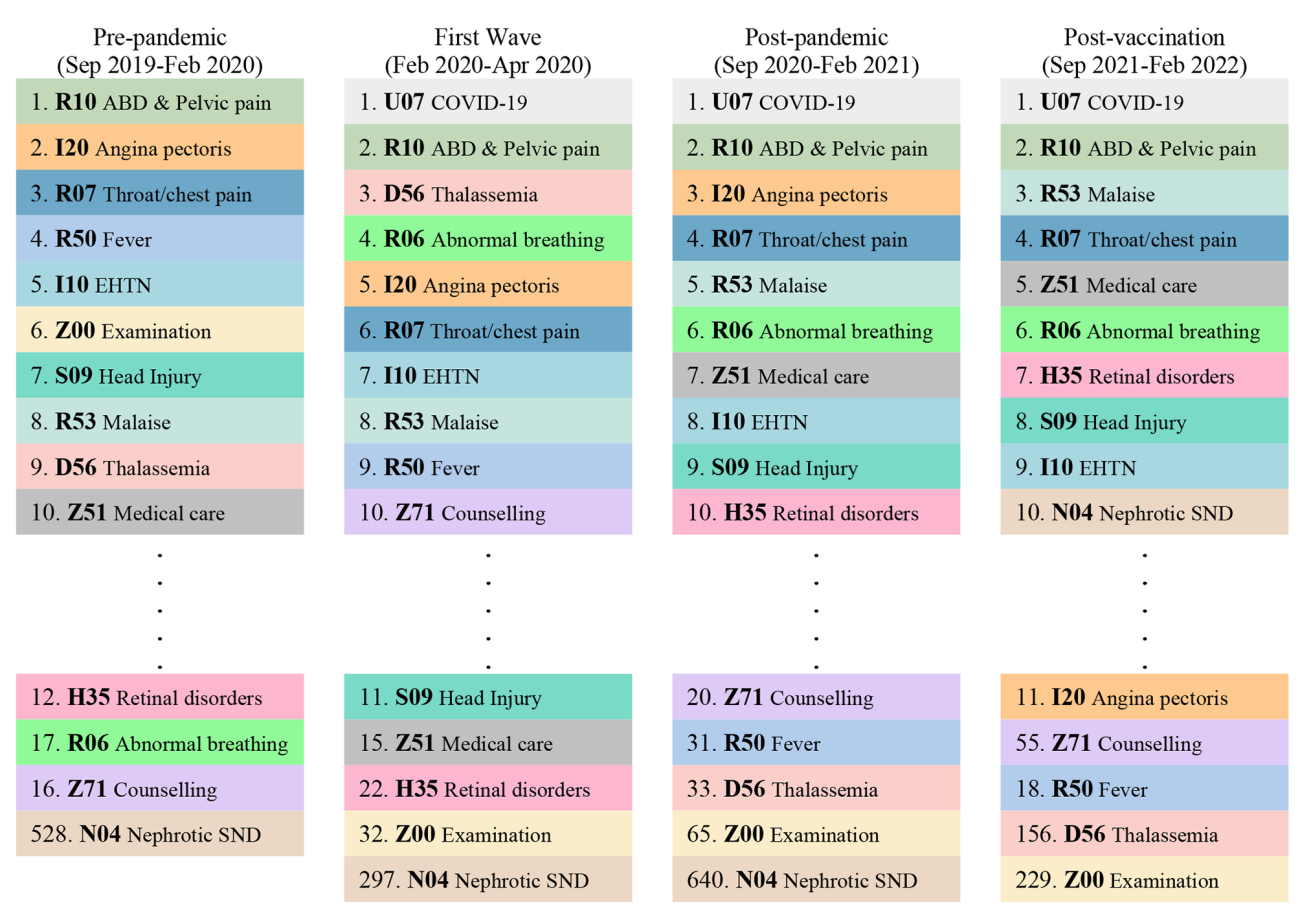
Ten most frequent ICD-10 diagnosis codes for emergency department registration during the COVID-19 pandemic. ABD & Pelvic pain: Abdominal and Pelvic pain, EHTN: Essential (primary) Hypertension, Malaise: Malaise and Fatigue, Nephrotic SND: Nephrotic Syndrome

The first ten frequent ICD-10 codes in ED registration are displayed in Fig. 1. The most common reason for visiting the ED in the pre-pandemic period was abdominal and pelvic pain (5.9%). COVID-19 was the leading reason for visits since its outbreak, followed by abdominal and pelvic pain. After the COVID-19 outbreak, general examination visits decreased from 6,077 to under 700 in the post-pandemic and post-vaccination period. A thalassemia diagnosis was recorded in 5,430 visits before the pandemic, but in the following year and in two years it decreased to 1,454 and 352 respectively.

**Fig. 2:**
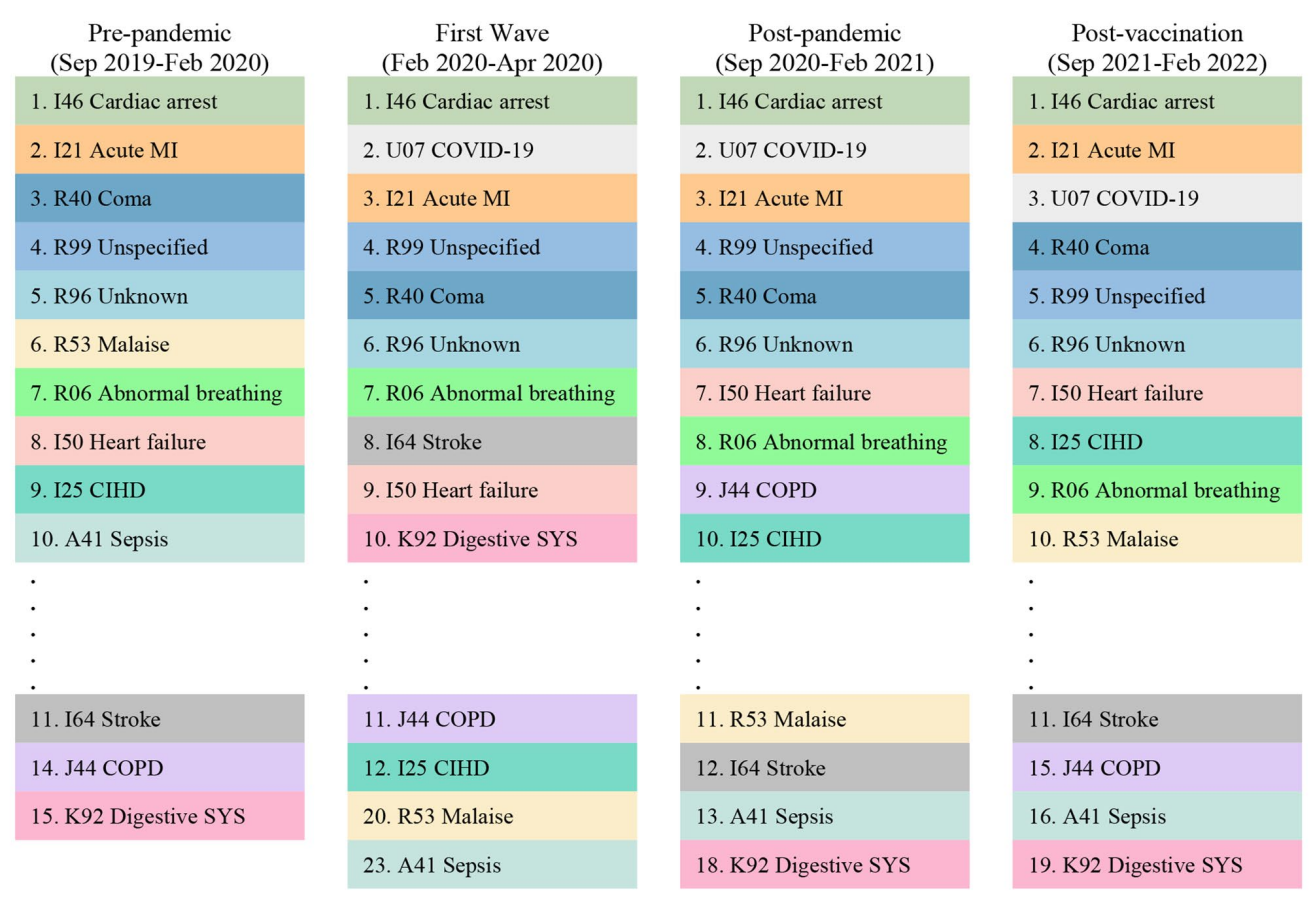
Ten most frequent ICD-10 diagnosis codes leading to death in the emergency department during the COVID-19 pandemic. MI: Myocardial Infarction, COPD: Chronic Obstructive Pulmonary Disease, Coma: Somnolence, Stupor and Coma, CIHD: Chronic Ischemic Heart Disease, Digestive SYS: Diseases of Digestive System, Malaise: Malaise and Fatigue

The pre-pandemic ED mortality risk for non-COVID-19 diseases was 8.17 per 1,000 visit-month, rising to 12.80 during the first wave of the pandemic (RR 1.57, [1.49–165]). During both the post-pandemic and post-vaccination periods, mortality risk was significantly higher than it was pre-pandemic, at 13.53 (RR 1.66, [1.59–1.72]) and 10.38 (RR 1.27, [1.22–1.32]) respectively. Table 3 provides details of mortality risks and RR. Figure 2 shows the ten most frequent diagnoses leading to ED mortality for each time period.

**Table 3:** Non-COVID-19 risk of emergency department mortality (per 1,000 visit-month) and Relative Risk (RR) of COVID-19 pandemic periods compared to the pre-pandemic baseline

	Pre-pandemic Sep 2019-Feb 2020	First Wave Feb-Apr 2020					Post-pandemic Sep 2020-Feb 2021					Post-vaccination Sep 2021-Feb 2022				
	Risk (Reference)	Risk	RR	Confidence Interval		p-value	Risk	RR	Confidence Interval		p-value	Risk	RR	Confidence Interval		p-value
Total	8.17	12.80	1.57	1.49	1.65	< 0.01	13.53	1.66	1.59	1.72	< 0.01	10.38	1.27	1.22	1.32	< 0.01
Age Group																
< 18	2.47	4.54	1.84	1.45	2.34	< 0.01	4.12	1.67	1.39	2.01	< 0.01	3.65	1.48	1.25	1.76	< 0.01
18–44	2.12	3.12	1.48	1.24	1.76	< 0.01	3.87	1.83	1.61	2.08	< 0.01	3.15	1.49	1.32	1.68	< 0.01
45–64	7.67	12.51	1.63	1.47	1.81	< 0.01	12.70	1.65	1.53	1.79	< 0.01	10.19	1.33	1.23	1.43	< 0.01
> 64	20.42	32.47	1.59	1.49	1.70	< 0.01	31.76	1.56	1.48	1.63	< 0.01	24.84	1.22	1.16	1.28	< 0.01
Gender																
Female	6.86	11.17	1.63	1.50	1.77	< 0.01	11.06	1.61	1.52	1.71	< 0.01	8.33	1.21	1.14	1.29	< 0.01
Male	9.44	14.25	1.51	1.41	1.62	< 0.01	15.83	1.68	1.59	1.76	< 0.01	12.37	1.31	1.25	1.38	< 0.01

### CVD

During the pre-pandemic period, the ED visit incidence rate for cardiovascular diseases was 167.59 per million person-month. CVD visit incidence rate declined to 117.75 in the first wave and 140.19 in the post-pandemic. The CVD incidence rate was 168.17 in the post-vaccination period. The IRR estimated with negative binomial regression shows that there was no significant CVD visit incidence rate change during the first wave, post-pandemic, or post-vaccination periods comparatively. A diagnosis of angina pectoris and essential hypertension was the most frequent reason for ED visits in Iranian hospitals associated with CVD. Furthermore, CVD was the most common cause of death in the ED and cardiac arrest was the leading cause in all time periods. Acute myocardial infarction, heart failure, chronic obstructive pulmonary disease, and stroke were the other deadly conditions.

### Mental health and substance use disorders

The pre-pandemic incidence rate of mental health and substance use disorders was 8.18 per million person-month. The visit incidence rate declined to 4.57 (IRR 0.53, [0.32 to 0.90]) in the first COVID-19 wave and to 4.75 (IRR 0.64, [0.43 to 1.97]) in the post-pandemic period. In the post-vaccination period, the mental health and substance use disorders visit rate was 9.41 with no significant change compared to pre-pandemic (IRR 1.27, [0.86–1.88]).

### Self-harm

For the pre-pandemic, first wave, post-pandemic, and post-vaccination periods, visit incidence rates related to self-harm were respectively 0.92, 1.11, 1.28, and 1.44 per million person-month. As compared to before the pandemic, there was no significant change in self-harm visit rate.

### Unintentional injuries

The unintentional injuries incidence rate of ED visits was 10.19 in pre-pandemic, 11.49 in first wave and 10.47 per million person-month in post-pandemic periods. The visit incidence rate of unintentional injuries increased significantly after vaccination compared to that of the pre-pandemic period (IRR 1.63, [1.30–2.03]).

## Discussion

For this cross-sectional study, we investigated the changes in non-COVID-19 ED visits and mortality for about 41 million Iranians during three different time periods after the COVID-19 outbreak. The first SARS-CoV-2 infection in Iran was confirmed on February 19, 2020. The first wave of COVID-19 with full restriction rules was from February 20 to April 19, 2020. During this wave, the incidence rate of non-COVID-19 ED visits decreased by 32% in all age groups, especially children, which is consistent with results from previous studies [28, 30, 33, 34, 38, 39, 60, 81–83].

The COVID-19 pandemic affected ED visits in many ways. The first was the direct effect of the pandemic, which caused more crowding in ED departments and longer wait times for non-COVID-19 patients. This was especially evident in Iran, where COVID-19 diagnostic tests were more affordable in public hospitals than in other laboratories and test centers [84–86]. As a result of lockdown measures, fewer people were able to reach large and specialized urban hospitals [87, 88]. This was particularly concerning in Iran, where the unequal distribution of healthcare resources across provinces often necessitates travel to more developed areas for medical treatment [89–94]. Therefore, lockdowns greatly impact healthcare access [38].

On the service demand side, fear of SARS-CoV-2 infection, as well as uncertainty about resource availability, led patients to avoid the hospital. Because hospital EDs never closed, unlike departments conducting elective surgical activities, ED visits were mostly influenced by demand, rather than supply. Some studies have shown that even in cities where COVID-19 had a low incidence rate and did not disrupt services, fewer people visited the ED than before the pandemic [28, 29].

One qualitative study suggested that patients avoid visiting the hospital ED not only for fear of infection, but also for fear that resources will not reach sick patients with COVID-19 [10]. Patient separation from family and friends was another reason for postponing visits during social distance periods [10]. With many physician offices and clinics closed during the first wave in Iran, the decrease in hospital ED visits is concerning. The home health-care system and e-health technologies were not well developed in Iran at the time, and patients may not have received the needed services [95–97]. According to some studies, avoidable ED visits decreased more than essential visits did [31, 32, 98]. In this study, it was not possible to distinguish between necessary and unnecessary visits. However, we found that “general examination” was no longer present in the first ten emergency diagnoses after the COVID-19 pandemic.

During the post-pandemic period, from September 22 to February 18, 2021, 27% of non-COVID-19 ED visits decreased compared to those in the same period pre-pandemic. According to these data, the decrease in visits did not recover after a year. Some studies have confirmed this result [34, 98]. However, in some countries, the decrease in ED visits was only observed during the first wave of COVID-19 and was recovered in the time afterward [33, 39, 82].

Additionally, this study examined non-COVID-19 ED visits during the second pandemic year, from September 23, 2021, to February 19, 2022. During this time, the incidence rate of visits in Iran increased by 11.3% following COVID-19 vaccinations being made available. It may be because people felt safer after being vaccinated and decided to reinitiate postponed health care. Because the SARS-CoV-2 Delta variant wave in Iran was severe, the increased ED visitation may be linked to a post-acute sequelae SARS-CoV-2 infection or long COVID [40, 99, 100].

ED visits were also affected by the return to normal routines, such as an increase in the number of trips and resumed business activities. A significant increase in the number of unintentional injuries was observed after all social distance restrictions were lifted and businesses reopened. This result has been reported in similar studies [43, 61, 62]. Even the adverse effects of the COVID-19 vaccine may have caused ED visits [101, 102].

Changes in the incidence rate of ED visits for cardiovascular diseases during the pandemic were insignificant, which is contrary to other studies that reported a decrease in visits [46, 103]. Additionally, in contrast to studies conducted in other countries, this study found that during the first year of the pandemic, incidences of ED visits for self-harm and unintentional injuries did not change compared to the pre-pandemic period [36, 42, 44, 47, 60]. It was found that the mental health and substance use disorders visit incidence rate declined after COVID-19 in this study, contrary to the findings of many other studies[52–57]. However, the unspecified diagnoses in ED registrations should also be taken into account when analyzing cause-specific visits.

Among the important findings of this study is the decline in thalassemia-related ED visits. Iran is one of the countries located on the thalassemia belt with a large thalassemia population. In public hospitals, thalassemia patients receive low-cost healthcare, including blood transfusions and iron chelation therapy. The import of critical pharmaceuticals for these patients was disrupted many times in Iran [104, 105]. The issue can contribute to other problems such as limiting access to healthcare during lockdowns and decreasing visits due to virus fears [106]. As a result, thalassemia ED visits decreased during the COVID-19 pandemic, which requires further investigation.

The results have shown that the mortality risk of non-COVID-19 ED visits after the pandemic increased during all time periods compared to that of the pre-pandemic period. Emergency rooms appeared to be busy with critical patients, whereas noncritical patients preferred to wait for services or receive them somewhere other than in the hospital. Some studies found similar results [31, 38, 60]. According to this study, CVD-related health conditions were among the leading causes of ED mortality. This finding is in line with Iran’s high CVD mortality rate [107, 108].

As a final note, not all changes in health outcomes during the pandemic can be attributed to COVID-19 disease directly or indirectly. Over time, many factors, such as health policy changes, economic conditions, and social factors, may have influenced hospital ED visits. A detailed study of changes in hospital visits and outcomes in non-COVID-19 disease groups during the COVID-19 pandemic will alert policy makers to the indirect and long-term effects of this epidemic. While the COVID-19 pandemic no longer qualifies as an emergency, it is crucial to consider its effects on recovery in the post-pandemic period as well as planning for future crises. Clearing and unifying data, not only at the hospital level, but in all providers and all financing agents can assist in monitoring and evaluating the situation and examining the relevant challenges. Developing countries should seriously consider this case into account and prioritizing big data capabilities within their health systems.

### Limitation

This study had two main limitations. The first was that we could not consider a longer period before the pandemic. This is because the integrated hospital databases in IHIO were launched just a few months before the COVID-19 outbreak. To overcome this limitation, we examined the same months in the following two years to consider seasonal changes.

The second issue was not separating emergency visits into necessary and unnecessary or avoidable and unavoidable. The ED registration data in this study do not include patient triage. The IHIO claims data contain only information about health conditions classified using the ICD codes for the 10th edition of the ICD.

## Conclusion

In this study, we investigated the changes in ED visits and associated mortality during the COVID-19 pandemic in 956 hospitals. Our analysis revealed significant shifts in non-COVID-19 ED visits and mortality rates over the pandemic. During the first year of the pandemic, we observed a substantial decline in non-COVID-19 hospital ED visits, which coincided with the national lockdown. However, as the pandemic progressed and COVID-19 vaccination became available, ED visit rates increased again in the second year.

Importantly, our study revealed a concerning trend of increased non-COVID-19 ED mortality risk throughout the pandemic. The risk of mortality for non-COVID-19 patients rose significantly during the first wave of COVID-19 and remained high even two years after the outbreak. This underscores the long-lasting impact of the pandemic on non-COVID-19 health outcomes. Our findings contribute to the existing literature by shedding light on the indirect effects of the COVID-19 pandemic on non-COVID-19 hospital ED visits and mortality in Iran. It is crucial for healthcare systems to recognize and address the challenges posed by the COVID-19 pandemic to non-COVID-19 health conditions.

## Data Availability

According to Iran health insurance organization privacy rules, the source data cannot be accessed publicly. In case of further questions, please contact razimoghadam.mahya@gmail.com.

## Abbreviations

ED: Emergency Department
IHIO: Iran Health Insurance Organization
IRR: Incidence Rate Ratio
RR: Relative Risk
MI: Myocardial Infarction
COPD: Chronic Obstructive Pulmonary Disease
CIHD: Chronic Ischemic Heart Disease
Digestive SYS: Diseases of Digestive System
ABD & Pelvic pain: Abdominal and Pelvic pain
EHTN: Essential (primary) Hypertension
Nephrotic SND: Nephrotic Syndrome
